# TRPV2-induced Ca^2+^-calcineurin-NFAT signaling regulates differentiation of osteoclast in multiple myeloma

**DOI:** 10.1186/s12964-018-0280-8

**Published:** 2018-10-16

**Authors:** Hua Bai, Huayuan Zhu, Qing Yan, Xuxing Shen, Xiupan Lu, Juejin Wang, Jianyong Li, Lijuan Chen

**Affiliations:** 1 Department of Hematology, First Affiliated Hospital of Nanjing Medical University, Jiangsu Province Hospital, No. 300 Guangzhou Road, Nanjing, 210029 Jiangsu Province China; 2 Department of Physiology, Nanjing Medical University, Nanjing, 211166 Jiangsu China

**Keywords:** Myeloma bone disease, TRPV2, Calcium, Osteoimmunology, Osteoclastogenesis

## Abstract

**Background:**

Myeloma bone disease (MBD) can cause bone destruction and increase the level of Ca^2+^ concentration in the bone marrow microenvironment by stimulating osteoclastic differentiation. Nevertheless, the relationships between MBD and highly efficient stimuli of Ca^2+^ in multiple myeloma (MM) progression, and possible regulatory mechanisms are poorly defined. Here, we reported that the nonselective cation channel transient receptor potential vanilloid 2 (TRPV2) plays a functional role in Ca^2+^ oscillations and osteoclastogenesis.

**Methods:**

To investigate the expression of TRPV2 in MM, we analyzed publicly available MM data sets and performed immunohistochemistry in MM patients. The correlations between TRPV2 expression levels and osteoclast-related cytokines were analyzed. Fluo-4 staining and ELISA assays were used to assess the regulated function of TRPV2 in intracellular Ca^2+^ and cytokines. Western blotting and Chromatin immunoprecipitation (ChIP) assays were performed to explore the signaling pathway of TRPV2-induced osteoclastic differentiation. Real-time PCR, Western blotting, ELISA and tartrate-resistant acid phosphatase (TRAP) staining were performed to detect the biological effects of TRPV2 inhibitor on osteoclastogenesis.

**Results:**

The functional expression of TRPV2, involved in the osteolysis through gating the calcium influx, was changed in the MM cells cultured in a high Ca^2+^ environment. Mechanistically, TRPV2 modulates nuclear factor-κB ligand (RANKL)-dependent osteoclastic differentiation through the Ca^2+^-calcineurin-NFAT signaling pathway. Of clinical relevance, systemic administration with SKF96365 could attenuate the MM-induced osteoclast formation in vitro.

**Conclusions:**

Our study uncovers the possible roles of TRPV2, which enhances MBD, suggesting that targeting osteocyte-MM cells interactions through blockade of TRPV2 channel may provide a promising treatment strategy in MM.

**Electronic supplementary material:**

The online version of this article (10.1186/s12964-018-0280-8) contains supplementary material, which is available to authorized users.

## Background

Approximately 80% patients with multiple myeloma (MM) present with bone lesions, hypercalcemia, fractures or bone pain during the course of disease [1, 2]. The abnormal calcium reabsorption in renal tubules leads to hypercalcemia in myeloma, which induces the dysregulated bone remodeling [3–5], and Ca^2+^ ions are directly released into the bone matrix during bone remodeling [6]. Ca^2+^ concentration is found to be elevated in the serum and bone marrow microenvironment of MM patients, which is positively correlated with myeloma bone disease and hypercalcemic crisis. Moreover, the elevated Ca^2+^ accelerates myeloma bone destruction and reabsorption through MM-osteoclast (OCL) interactions [7, 8]. However, it is not clear whether MM cells under high level of extracellular calcium concentration ([Ca^2+^]_o_) could regulate the differentiation of osteocytes under exposure to in the bone marrow microenvironment surrounding bone destruction.

Intracellular Ca^2+^ ([Ca^2+^]_i_) could act as an secondary messenger involved in multiple cellular functions, including inflammation, molecular transportation and gene transcription [9]. In different cells, the plasma membrane Ca^2+^-permeable channels are involved in Ca^2+^ influx [10, 11]. Calcium-sensing receptor (CaSR) was activated by extracellular Ca^2+^, which promoted bone metastasis in renal cell carcinomas [12]. Moreover, many Ca^2+^ channels might associate with osteoclasts,and highlights that the locally increased extracellular Ca^2+^ could induce osteoclastic differentiation [13]. However, whether myeloma cells possess the ability to response the changes in extracellular calcium concentration, and which [Ca^2+^]_i_ signaling pathways involved in high Ca^2+^-induced osteoclastic differentiation in MM have not been fully elucidated.

One candidate for a Ca^2+^-sensing channel that could be expressed on MM cells is the transient receptor potential vanilloid type 2 [14], which is Ca^2+^ permeable channel contributing to calcium homeostasis [15]. TRPV2 is widely expressed in different organs and tissues [16]. Notably, the expression of TRPV2 was up regulated in MM patients [17]. Since TRPV2 is also highly expressed in MM cells [18], we assume that TRPV2 might act as a mediator to transmit Ca^2+^ into MM cells.

In this study, we reported that the activation of TRPV2 by high [Ca^2+^]_o_ increases the osteoclastic activity. Mechanistically, TRPV2-induced Ca^2+^ influx modulates calcineurin**-**NFAT activity and mediates the release of RANKL in MM cells. This investigation of RANKL-mediated osteoclastic differentiation via TRPV2 in MM cells may shed a light for the treatment of myeloma bone disease.

## Methods

### Clinical samples and cells

90 newly diagnosed MM patients were recruited from January 2013 to March 2016 in the First Affiliated Hospital of Nanjing Medical University. MM was diagnosed according to the 2008 World Health Organization (WHO) criteria. All medium were supplemented with 10% fetal bovine serum (Gibco, USA). All cells were maintained in a 5% CO_2_ cell culture incubator. Cells were transfected with different lentiviral constructs (carrying whole *TRPV2* transcript or an empty negative control vector) and harvested at day3 post-transfection for analysis. Cells were transfected with a siRNA for TRPV2 (Ribobio Technologies) or scrambled siRNA as a negative control using lipofectamine 3000. Transduction efficiency was determined by Western blotting.

### Gene expression profiling (GEP) and data analysis

Gene Expression Omnibus (GEO) data were carried out to examine the expression of transient receptor potential channels in MM patients (GSE24080) and MM cell lines (GSE6205) [19]. Data acquisition and normalization methods in these datasets have been described previously [19]. The mRNA expression of *TRPV2* (GSE2658) in plasma cells was determined using the Affymetrix U133Plus2.0 microarray (Affymetrix, USA), which were performed as previously described [20].

### Western blotting and quantitative real-time PCR (qRT-PCR) analyses

Protein extracts or nuclear protein extracts were electrophoresed on polyacrylamide gels, blotted, and then incubated with primary antibodies overnight (4 °C). Lamin B1 and NFATc3 (Proteintech, USA); MMP-9, P53, GAPDH and β-actin (Cell signaling Technology, USA); TRPV2 (Alomone, Israel); Cathepsin K (Bioworld, USA); calcineurin (Cusabio, China); secondary antibodies (Vazyme Biotech, China), and then developed.

Total RNA from cell lines was isolated and supplied to reverse transcription; qRT-PCR was done using a StepOnePlus RT-PCR System (Applied Biosystems, USA). GAPDH levels were used to normalize all genes expression levels. The sequences of primers were listed as following (5′-3′): *GAPDH*, sense, TTTGGTATCGTGGAAGGAC, antisense, AAAGGTGGAGGAGTGGGT; mice *Cathepsin K*, sense, GCGTTGTTCTTATTCCGAGC, antisense, CAGCAGAGGTGTGTACTATG; mice *MMP-9*, sense, GCTGACTACGATAAGGACGGCA, antisense, GCGGCCCTCAAAGATGAACGG; human *RANKL*, sense, AAGGAGCTGTGCAAAAGGAA, antisense, CGAAAGCAAATGTTGGCATA; *TRPV2*, sense, GGAGGAAGACAGGACCCTTGACA, antisense, TTCCCTTTCGGTAGTTGAGGTTGA; mice *GAPDH*, sence, AACGACCCCTTCATTGACCT, antisense, CACCAGTAGACTCCACGACA.

### Immunohistochemistry and ELISA

BM tissues were harvested and fixed in 10% formaldehyde, and antigen retrieval was processed in EDTA-containing antigen retrieval buffer (pH = 8.0) in 95 °C,and followed by 3% H_2_O_2_ incubation for 30 min. Next,the samples were blocked by goat serum for 10 min, and incubated with Anti-TRPV2 antibody over night at 4 °C, the secondary antibody was incubated for 30 min before visualization by DAB reagent.

Double-staining fluorescent immunohistochemistry was performed on fixed MM cells, and processed for Anti-TRPV2 antibody and Anti-CD38 antibody (Proteintech, USA). After incubation and wash, cells were incubated with secondary antibody conjugates (Invitrogen, USA) and DAPI was used to counterstain the nuclei.

TNF-α, IL-1β and RANKL in conditioned media were quantified by commercially available ELISA kits (Yifeixue Bio Tech, China), per the manufacturers’ instructions.

### Chromatin immunoprecipitation

Chromatin immunoprecipitation (ChIP) assays were done using the ChIP Assay kit (Beyotime, China) according to the manufacturer’s instructions. Briefly, MM cells were cultured with [Ca^2+^]_o_ or SKF96365 for 24 h. Treated cells were cross-linked with 1% formaldehyde in PBS for 10 min. Next, immunoprecipitation was utilized with NFATc3 antibody (5 μg) (Proteintech, USA) or IgG as negative control at 4 °C overnight. Protein A + G were used for pulling down immune complexes. Then, the complexes were washed out with elution buffer (1% SDS, 0.1 mol/L NaHCO_3_), and cross-linking reversed with NaCl at 65 °C for 4 h. DNA was purified and carried out with PCR amplification of the human *RANKL* promoter (− 1524 to − 1324 bp) using the following primers (5′-3′): sense, GATACACATATAAATGCTAA, antisense, CGCTAATGAGTATTTCTCTA. The results of RT-PCR were analyzed by image J.

### Osteoclastic differentiation assays in vitro

RAW264.7 cells (a mouse macrophage cell line obtained from Keygene) were cultured with MM cell conditioned medium contained αMEM (ratio 1:1) or 10% fetal bovine serum contained αMEM in each experiment [21–24]. On the day of harvest, Raw264.7 cells were stained for TRAP using an Acid-phosphatase leukocyte staining kit (Sigma-Aldrich, USA). Osteoclasts were identified and enumerated under microscopy as TRAP^+^ cells with ≥3 nuclei/cell.

### Measurement of intracellular Ca^2+^ and serum calcium

AND LP-1 cells cultured in confocal dishes (Thermo Fisher, USA) were first loaded with 5 μM Fluo 4-AM in Hanks’ Balanced Salt Solution (HBSS) (final concentration of DMSO, 0.1%) at room temperature for 30 min protected from light, then washed twice in HBSS at room temperature. Image analysis was performed using Zen2011 software, and fluorescence of every cell in each field was measured. A Direct-reading ISE (ion-selective electrodes) analyzer was used for iCa^2+^ measurement in serum samples from randomly selected patients.

### Statistical analysis

Data are presented as mean ± SEM and analysis involved use of GraphPad Prism 5 (GraphPad Software, Inc., USA). Statistical analyses were performed using Student’s t-test or one-way analysis of variance (ANOVA). *P <* 0.05 was considered statistically significant.

## Results

### Higher TRPV2 expression predicts poor prognosis in MM patients

**Fig. 1:**
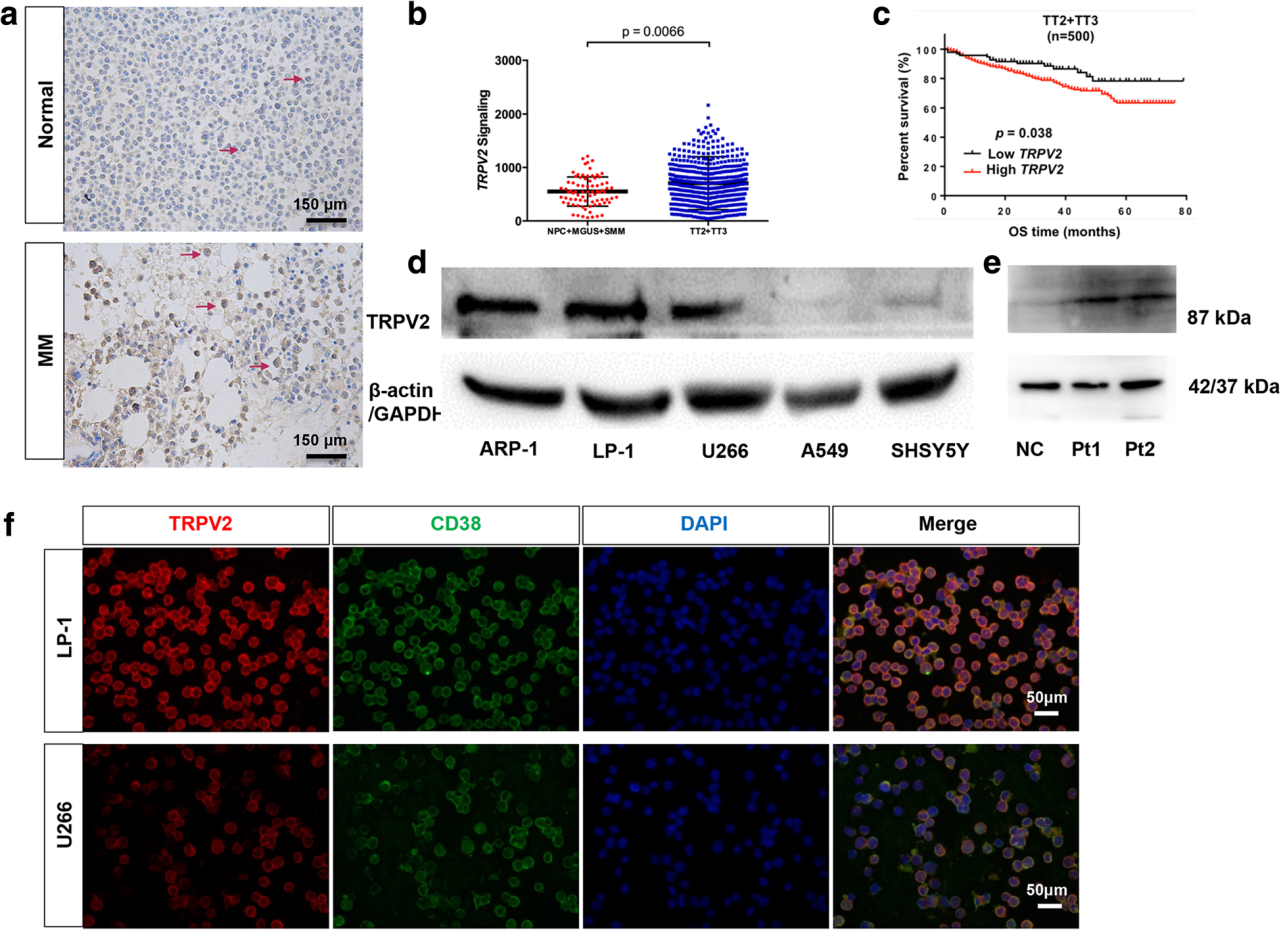
TRPV2 is highly expressed in MM patients and associated with poor prognosis. **a** Representative image of TRPV2 expression in NC and MM BM by immunehistochemical staining. **b** *TRPV2* channel expression levels in NP + MGUS+SMM and MM from GSE5900 and GSE2658. **c** Kaplan-Meier analysis and log-rank tests were used to evaluate whether *TRPV2* expression level was associated with OS in TT2 + TT3 trial (*P* = 0.038, *n* = 500). **d** and **e** Western blot detection of TRPV2 in MM cell lines, A549 and SHSY5Y cells (negative control), MM patients cells and normal donor cells (MNCs). **f** Double-staining Immunofluorescence detection showing TRPV2 (red) and CD38 (green) in MM cells. *P < 0.05

To assess the expression of TRPV2 channels in MM patients, we examined the protein expression of TRPV2 in bone marrow biopsy specimens from normal or MM bone marrow by immunohistochemistry. TRPV2 was upregulated in MM bone marrow compared to normal bone marrow (Fig. 1a and Additional file 1: Figure S1b). We also analyzed public gene expression data of bone marrow plasma cells from MM counterparts. In GSE24080, transcriptional level of TRPV2 in plasma cells of patients with shorter Event Free Survival (EFS, < 24 months) was significant higher as compared to patients with longer EFS (≥ 24 months) (Additional file 2: Figure S2a). Moreover, TRPV2 was overexpressed in patients with inferior overall survival (OS, < 24 months) as compared to patients with favorable OS (≥ 24 months) (*P =* 0.07, Additional file 2: Figure S2b). In GSE5900 and GSE2658, TRPV2 is overexpressed in MM patients compared with other donors, which have no bone lesions (NP, MGUS and SMM), so it indicated us the correlation between TRPV2 and bone lesions in MM (Fig. 1b). Furthermore, in GSE2658, MM patients with higher *TRPV2* expression had shorter OS as compared to patients with lower *TRPV2* expression (TT2 + TT3, Fig. 1c), which suggests the expression level of TRPV2 might affect the outcome of MM patients. Taken together, these results indicated that TRPV2 is highly expressed in MM patients and correlated with poor prognosis and bone lesions.

Next, we explored mRNA expression of *TRPV2* in MM cell lines. The Cancer Cell Line Encyclopedia (CCLE) database shows that *TRPV2* expression is higher in MM cell lines, compared to other cancer cell lines, such as non-small cell lung carcinoma cells and neuroblastoma cells (Additional file 2: Figure S2 k). TRPV2 protein in MM cell lines ARP-1, LP-1 was obviously higher than U266, A549 and SH-SY5Y by Western blotting (Fig. 1d and Additional file 3: Figure S3 h). Moreover, we also examined the expression of TRPV2 in MM patients cells and normal donor cells (MNCs) by Western blotting in Fig. 1e, and TRPV2 protein was upregulated in MM patients cells. Presence of TRPV2 in MM cells was confirmed by immunofluorescence staining (Fig. 1f and Additional file 4: Figure S4a), both U266 and A549 were used as the negative control (Additional file 3: Figure S3 g and h), all three-cell lines have a high green fluorescence of CD38, but U266 and A549 have low red fluorescence of TRPV2, which indicated that TRPV2 is expressed at both mRNA and protein levels in MM cells.

### High [Ca^2+^]_o_ induces TRPV2 expression and enhances the secretion of osteoclast-related cytokines in MM cells

**Fig. 2:**
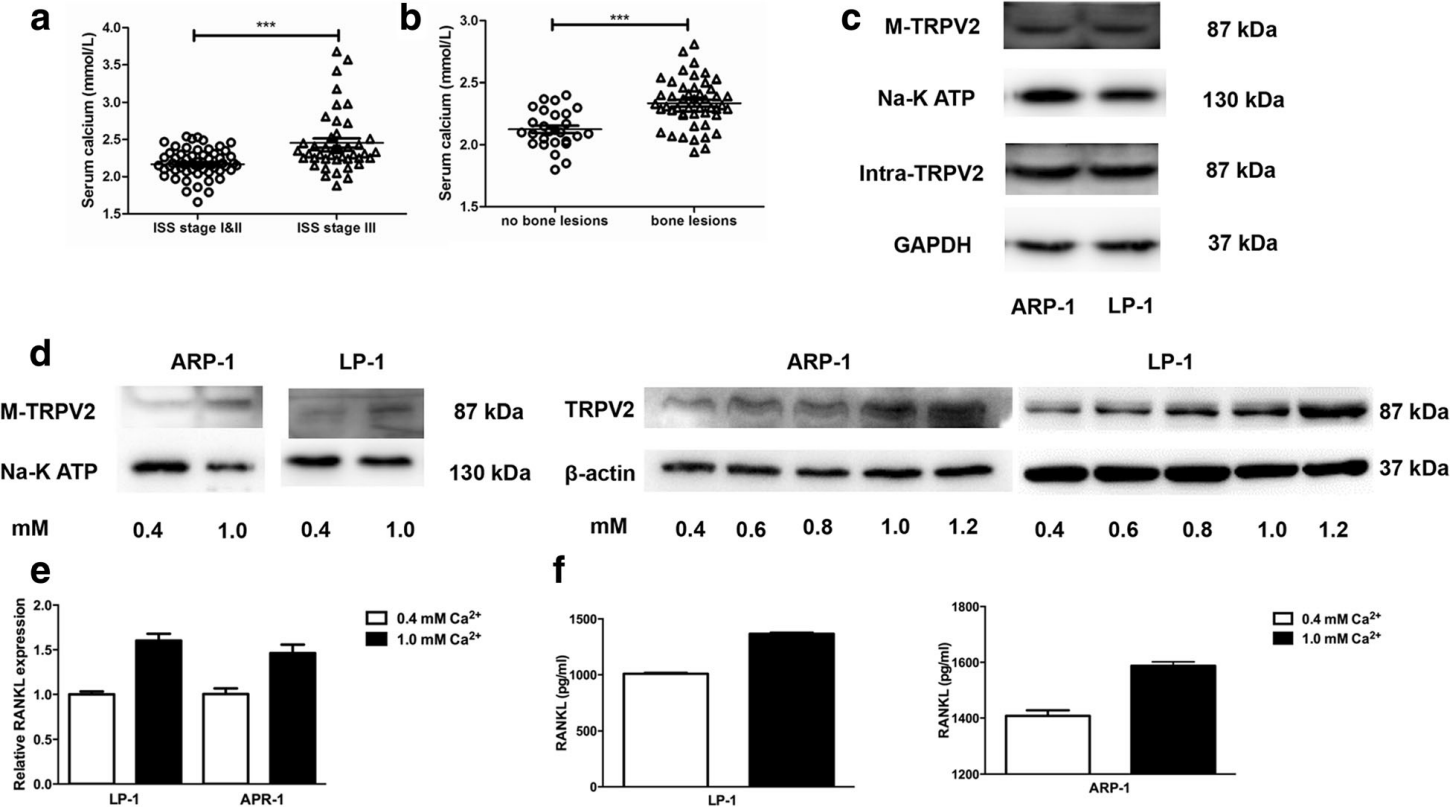
High [Ca^2+^]_o_ increases the expression of TRPV2 and the secretion of inflammatory cytokines in MM cells. **a** The levels of serum calcium in ISS stage III were significantly higher than those in patients with ISS stage I & II (*P* = 0.001). **b** The levels of serum calcium were significantly high in incipient MM patients with bone lesions (*P* = 0.0001). **c** Western blot analyses of TRPV2 expression variation in plasma membrane and intracellular levels. **d** Western blot analyses of plasma membrane and total TRPV2 expression variation in LP-1 and ARP-1 treated with a range of [Ca^2+^]_o_ concentrations. **e** and **f** qRT-PCR and ELISA analyses showing RANKL expression of MM cells incubated with a range of [Ca^2+^]_o_ concentrations. *P < 0.05; **P < 0.01; ***P < 0.001

Myeloma cells are exposed to high level of [Ca^2+^]_o_ with bone destruction [25]. Therefore, we examined the expression of serum calcium concentration in 90 MM patients, which was significantly higher in patients with International Staging System (ISS) stage III than ISS stages I & II (Fig. 2a), and the serum calcium was higher in MM patients with bone lesions (≥ 1) compared to those without lesions (Fig. 2b). Additionally, there were no associations between serum calcium and patient clinical baseline characteristics (such as gender and age), as well as other established prognostic factors (such as ESR, CRP, and LDH). However, serum calcium was correlated with sCr and ALB levels, respectively (Additional file 5: Table S1). Collectively, our results indicated the key role for calcium in the pathogenesis of MBD.

Next, we evaluated the effect of [Ca^2+^]_o_ on the cell viability upon different concentrations by using trypan blue staining and flow cytometric analysis, we found [Ca^2+^]_o_ could decrease cell viability after 7 days stimulation with 2.0, 2.8, 4.0, and 5.8 mM [Ca^2+^]_o,_ respectively. After treatment with 0.4–1.2 mM [Ca^2+^]_o_ for 12–48 h, the proliferation of myeloma cells remained unchanged at the concentration from 0.4 to 1.0 mM, but decreased significantly at 1.2 mM after 24, 36 and 48 h, respectively (Additional file 4: Figure S4a). Then, we investigated the level of plasma membrane expression of TRPV2 channels compare to the intracellular levels in MM cell lines ARP-1and LP-1, the results show that TRPV2 is expressed in both plasma membrane and intracellular (Fig. 2c). Notably, elevated protein levels of TRPV2 were detected after exposure to escalating concentration of [Ca^2+^]_o_ (Fig. 2d), suggesting that [Ca^2+^]_o_ might play role in regulating the expression of TRPV2.

Based on the correlation between [Ca^2+^]_o_ and TRPV2, we then utilized MM cells cultured in high calcium medium for further investigation. The previous studies reported the inflammatory cytokines, such as tumor necrosis factor (TNF)-α, interleukin (IL)-1β and nuclear factor-kappa B ligand (RANKL) promote osteoclastogenesis [26–28], here, we also found that the levels of RANKL were increased in a dose-dependent way after stimulation with 1.0 mM [Ca^2+^]_o_ (Fig. 2e and f). Surprisingly, RANKL showed the increasing tendency with TRPV2 in high [Ca^2+^]_o_ microenvironment. Taken together, high concentration of [Ca^2+^]_o_ could increase the expression of TRPV2 and stimulate the secretion of osteoclast-related cytokines in MM cells.

### TRPV2 regulates the secretion of RANKL via Ca^2+^-calcineurin-NFATc3 signaling pathway in MM cells

**Fig. 3:**
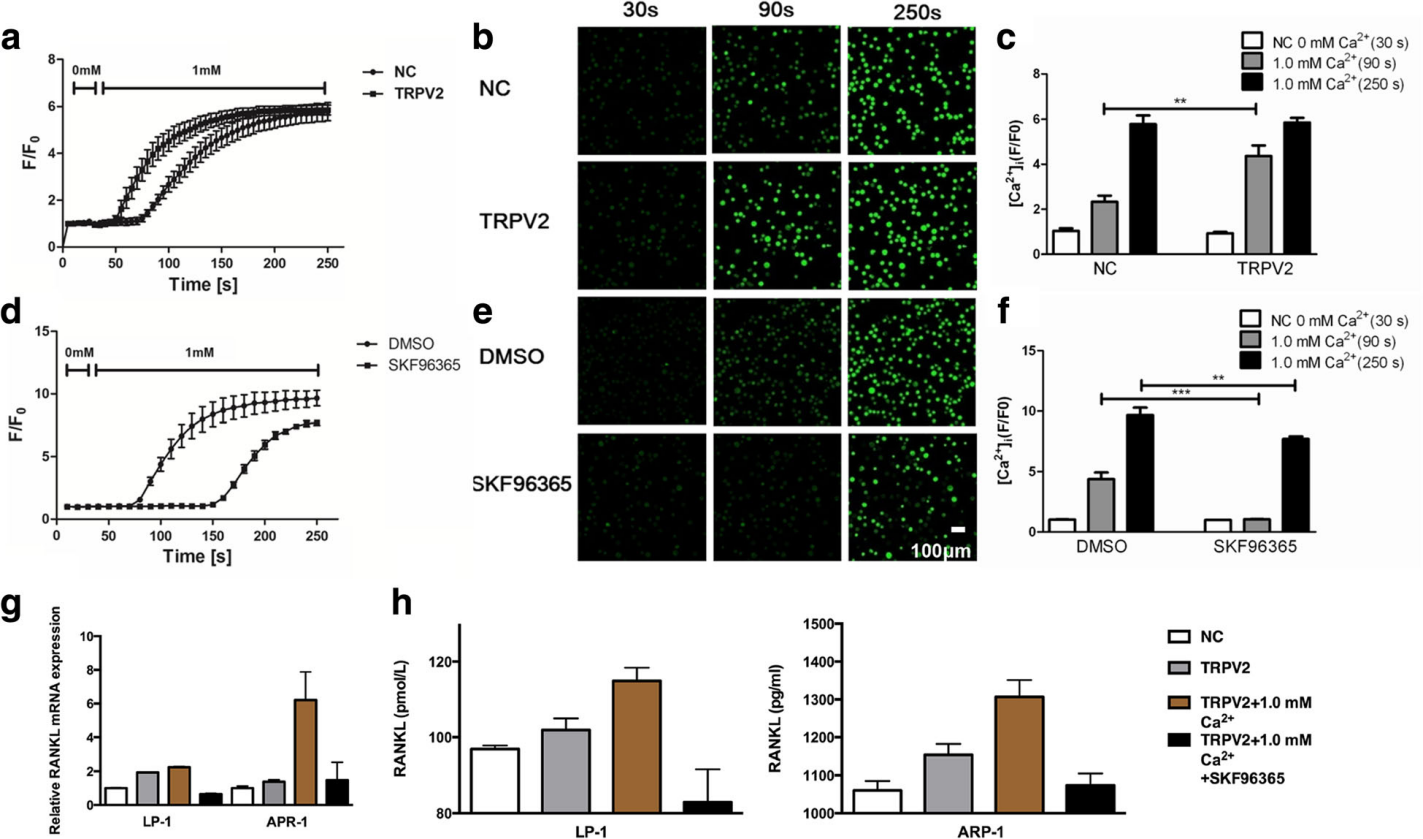
TRPV2 regulates the Ca^2+^ influx in MM cells. **a**-**c** Kinetic curves, images and mean data demonstrating that overexpressing TRPV2 channel enhances the [Ca^2+^]_o_-induced [Ca^2+^]_i_ elevation in LP-1. **d**-**f** Kinetic curves, images and mean data demonstrating that the inhibition of TRPV2 channel attenuates the [Ca^2+^]_o_-induced [Ca^2+^]_i_ elevation in LP-1. **g** and **h** qRT-PCR and ELISA analyses confirming the overexpression and inhibition of TRPV2 channel influence both basal expression and high [Ca^2+^]_o_-induced expression of RANKL. *P < 0.05; **P < 0.01; ***P < 0.001

Next, we investigated the possible mechanism how TRPV2 regulates the secretion of cytokines. First, we successfully overexpressed the level of TRPV2 in ARP-1 and LP-1 (Additional file 6: Figure S5b). To determine the contribution of TRPV2 to the [Ca^2+^]_o_ influx, we recorded the responses of LP-1 cells to rapid changes of [Ca^2+^]_o_ from a calcium free to a 1.0 mM [Ca^2+^]_o_ containing solution. LP-1 cells were transfected with TPRV2 or negative control vector, cultured in HBSS medium and exposure to a rapid increase of 1.0 mM [Ca^2+^]_o_ at 30 s (s), confocal microscopy showed that a transient and rapid raise of green fluorescence in TRPV2 overexpressed group, which was obviously higher than control at 90 s, and reached the same peak phase at 250 s (Fig. 3a-c). By contrast, we investigated whether the inhibition of TRPV2 channel acts as a controller on the entrance of [Ca^2+^]_o_. Typically, DMSO-treated cells sparkled than SKF96365-treated cells during 70s to 250 s and the ultimate strength also convinced the result (Fig. 3d-f). Above results demonstrated that [Ca^2+^]_o_ stimulation and TRPV2-induced regulation mediate the transient change in [Ca^2+^]_i_. Moreover, to further confirm functional effect of TRPV2 channel, RANKL expression induced by 1.0 mM [Ca^2+^]_o_ were detected by qRT-PCR and ELISA assays. As shown in Fig. 3g and h, overexpression of TRPV2 conferred an increasing secretion of RANKL rather than a decrease in SKF96365-treated groups.

**Fig. 4:**
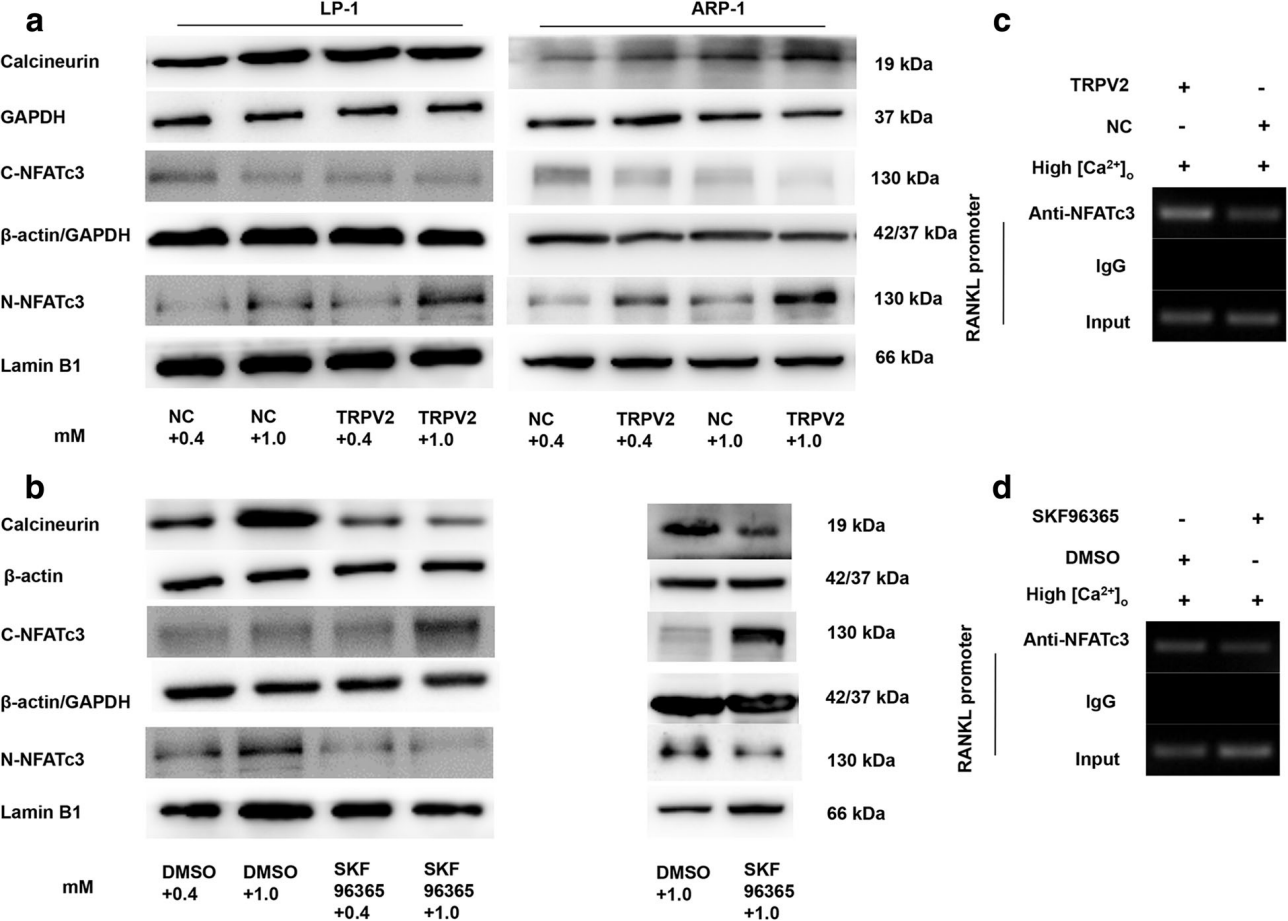
High [Ca^2+^]_o_-induced RANKL expression depends on [Ca^2+^]_i_/calcineurin/NFATc3 activation. The protein levels of calcineurin, nuclear NFATc3 (N-NFATc3) and cytosolic NFATc3 (C-NFATc3) of MM cells were measured by western blotting. **a** Cell fractions of ARP-1 and LP-1 over-expressed *TRPV2* were extracted and immunoblotted with antibodies. **b** Cell fractions of ARP-1 and LP-1 treated with SKF96365 were extracted and immunoblotted with antibodies. ChIP assays were performed to reveal high [Ca^2+^]_o_ induced *NFATc3* binding to the *RANKL* promoter. **c** *TRPV2* overexpression induced *NFATc3* binding to the *RANKL* promoter. **d** SKF96365 reduced the binding of *NFATc3* with *RANKL* promoter

Since inhibition of TRPV2 may reduce secretion of RANKL, we next assessed whether TRPV2 plays a role on the secretion of RANKL in MM cells. Consistent with our observations, myeloma cell could secrete RANKL [29], and high [Ca^2+^]_o_ increases secretion of RANKL through activation of calcineurin/NFAT signaling in osteoblasts [30], and previous studies showing that both N-terminal and C-terminal region of NFATc1/NFATc3 contain calcineurin binding site [31, 32]. To confirm that highly efficient stimuli of [Ca^2+^]_o_ activates the calcineurin/NFAT signaling in MM cells, we first examined the expression of calcineurin, the nuclear accumulation of NFATc3 (N-NFATc3) by Western blotting. Calcineurin and N-NFATc3 were increased with the treatment of 1.0 mM [Ca^2+^]_o_ (Fig. 4a), demonstrating that the stimuli of [Ca^2+^]_o_ could activate the calcineurin/NFATc3 signaling pathway in MM cells. Moreover, up-regulation of calcineurin and N-NFATc3 induced by 1.0 mM [Ca^2+^]_o_ was further enhanced by TRPV2 overexpression and could be reversed by SKF96365, respectively (Fig. 4a and b). These results suggested that TRPV2 might modulate the secretion of RANKL via Ca^2+^-calcineurin-NFATc3 signaling pathway in MM cells. We then investigated whether NFATc3 binds to presumptive binding element of *RANKL* by ChIP assays. Nuclear extracts from TRPV2 overexpressed LP-1 cells or SKF96365-treated LP-1 cells were utilized for immunoprecipitation with NFATc3 antibody. More *NFAT*c3 was bound to the promoter of *RANKL* in *TRPV2*-transfected cells as compared to NC (Fig. 4c). In contrast, SKF96365 notably reduced the binding of *NFATc3* to the *RANKL* promoter (Fig. 4d). These data revealed that *TRPV2* could activate *NFATc3,* which in turn bound to the *RANKL* promoter and induced at the transcriptional level.

Taken together, our results indicated that oscillations of [Ca^2+^]_i_ might be caused by the expression and functional change in TRPV2 channel and TRPV2 channel might contribute to the secretion of RANKL via calcineurin-NFATc3 signaling pathway in MM cells.

### The blockade of TRPV2 suppresses myeloma-induced osteoclastic differentiation in vitro

**Fig. 5:**
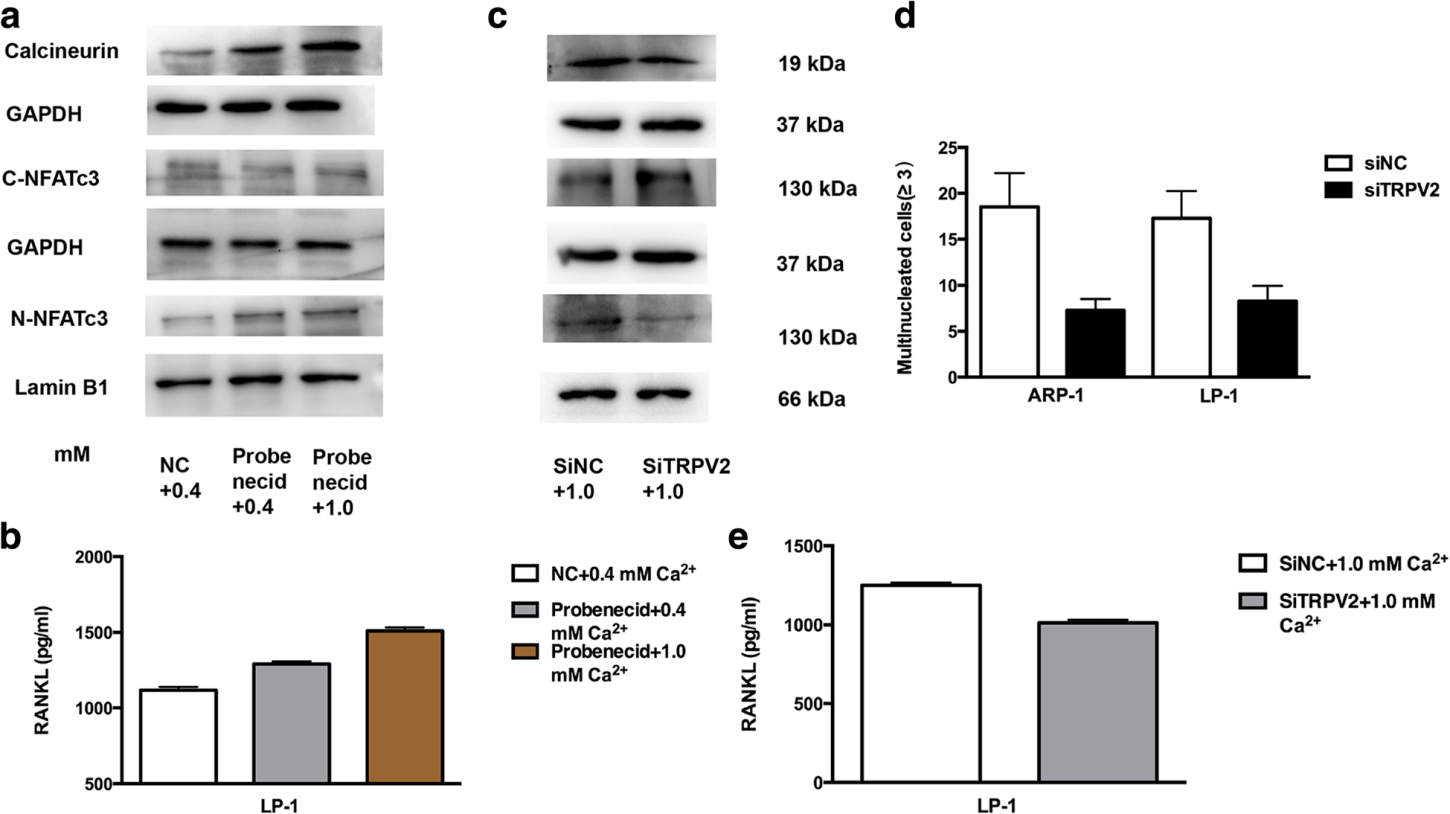
TRPV2 knockdown inhibits MM cells-induced osteoclastic differentiation. **a** The protein levels of calcineurin, nuclear NFATc3 (N-NFATc3) and cytosolic NFATc3 (C-NFATc3) of MM cells were measured by western blotting, cell fractions of LP-1 treated with Probenecid (1 uM) were extracted and immunoblotted with antibodies. **b** ELISA analyses confirming the treatment of Probenecid influence both basal expression and high [Ca^2+^]_o_-induced expression of RANKL. **c** The protein levels of calcineurin, nuclear NFATc3 (N-NFATc3) and cytosolic NFATc3 (C-NFATc3) of MM cells were measured by western blotting, cell fractions of TRPV2 knockdown cells were extracted and immunoblotted with antibodies. **d** TRAP staining and counts of multinucleated cells (≥ 3 nuclei/cell) after co-cultures with or without TRPV2 knockdown cells. **e** ELISA analyses confirming the treatment of TRPV2 knockdown influence high [Ca^2+^]_o_-induced expression of RANKL

**Fig. 6:**
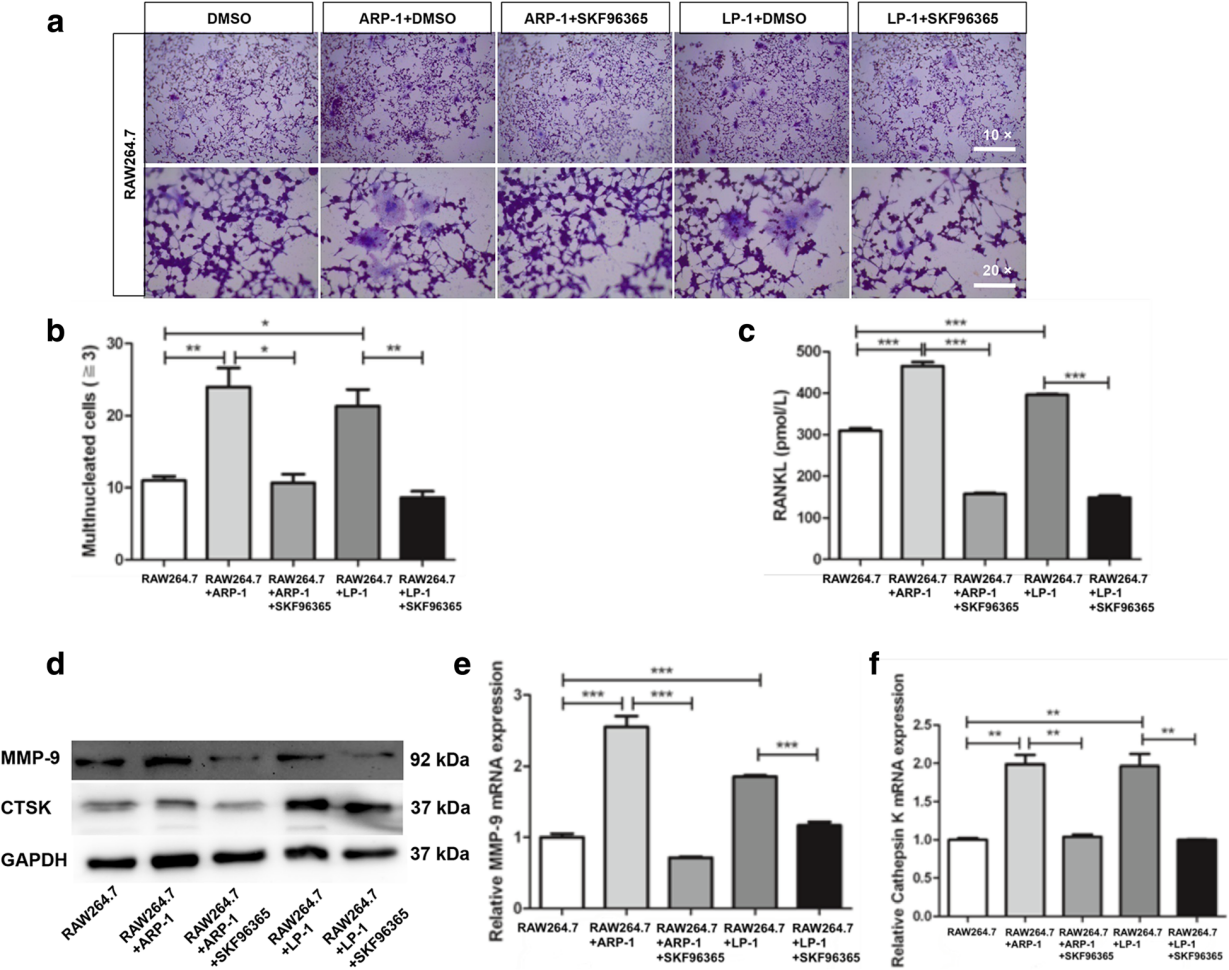
SKF96365 regulates MM cells-induced osteoclastic differentiation in vitro. **a** and **b** TRAP staining and counts of multinucleated cells (≥ 3 nuclei/cell) after co-cultures with or without SKF96365. **c** ELISA assays on RANKL protein expression in the co-cultured medium after co-cultures with or without SKF96365. **d**-**f** Western blotting and qRT-PCR measure of MMP-9 and CTSK expression variation in RAW264.7 cells. *P < 0.05; **P < 0.01; ***P < 0.001

To explore the capacity of TRPV2 agonist (Probenecid) in triggering the secretion of osteoclast-related cytokines. Firstly, we utilized Western blotting to investigate calcineurin/NFAT pathway, and our results suggested that Probenecid could trigger the activation of calcineurin/NFAT pathway, and 1.0 mM [Ca^2+^]_o_ could accelerated this tendency (Fig. 5a). Moreover, to further confirm functional effect of TRPV2 channel agonist, RANKL expression induced by 1.0 mM [Ca^2+^]_o_ and Probenecid were detected by ELISA assays (Fig. 5b). We utilized the SiRNA to knockdown TRPV2 expression in MM cells, and Ca^2+^-calcineurin-NFAT signaling is down-regulated in SiTRPV2 group compared to SiNC group (Fig. 5c and Additional file 6: Figure S5c). To investigate the possible role of TRPV2 knockdown in osteoclast differentiation, we co-cultured RAW264.7 with TRPV2-koncked down MM cells in high [Ca^2+^]_o_ DMEM medium, numbers of TRAP-positive multinucleated osteoclasts (MNCs) (≥ 3 nuclei/cell) in the RAW264.7-SiTRPV2 MM co-cultured group were significantly decreased (Fig. 5d and Additional file 4: Figure S4b), this result show the same tendency with RANKL expression (Fig. 5e), both of them confirm the effect of TRPV2 knock-down in osteoclast differentiation.To investigate the possible role of TRPV2 in bone destruction, we co-cultured RAW264.7 with MM cells in high [Ca^2+^]_o_ DMEM medium [33]. Osteoclastic differentiation of RAW264.7 treated with MM cells compared with RAW264.7 was evaluated in vitro. Numbers of TRAP-positive multinucleated osteoclasts (MNCs) (≥ 3 nuclei/cell) were generated by each and dramatically increased in RAW264.7-MM co-cultured cells compared with RAW264.7 cells (Fig. 6a). Surprisingly, we found the number of TRAP-positive MNCs and RANKL expression in the RAW264.7-MM co-cultured group were significantly decreased after the treatment of SKF96365, the results showed the same tendency with TRAP staining (Fig. 6b and c) [34]. As expected, western blotting and qRT-PCR showed that matrix metalloproteinase-9 (MMP-9) and cathepsin K (CTSK) were notably reduced in the SKF96365-treated cells (Fig. 6d-f). These results suggested that osteoclastic differentiation were increased in co-cultured cells and could be compromised by SKF96365.

## Discussion

The outcome of MM has been dramatically changed with the advent of new drugs such as bortezomib and lenalidomide. However, the agents for MBD such as saline replenishment and bisphosphonates could only partially postponed the advancement of osteolytic lesions [35], and the progression of MM related osteolytic lesions was just begun to be defined [36]. The destroyed bone remolding caused by the interaction in between myeloma cells and microenvironment cells was identified to play major role in pathogenesis of myeloma bone lesions [37, 38]. Here, we explored the possible role of TRPV2 channel in MM and identified its molecular mechanism in osteoclastic differentiation.

The activation of TRPV2 channel could increase the level of [Ca^2+^]_i_, which was involved in multifunctional processes, such as metabolism, molecular transport and gene transcription in tumor cells [39, 40], and TRPV2 channel was reported to be overexpressed in MM patients by microarray assay [17]. However, little was known about the channel function in MM, especially in MBD. In our study, we found overexpression of TRPV2 and serum calcium was correlated with poor prognosis in patients with MM. High concentration of [Ca^2+^]_o_ up-regulated TRPV2 channel expression in MM cells, the level of [Ca^2+^]_i_ was mainly increased via stimulation of Ca^2+^ influx transmitted by TRPV2 channel, while inhibition with SKF96365 almost abolished the effect of TRPV2 on [Ca^2+^]_i._. These results indicated a correlation between TRPV2 and Ca^2+^ in MBD.

Notably, TNF-α and IL-1β are well-identified bone-resorbing cytokines that may contribute to the development of the myeloma bone disease in MM [41, 42]. Both TNF-α and IL-1β could synergize with RANKL to induce osteoclastic differentiation [43, 44], TNF-α was found to induce osteoclast formation at multiple levels, not only stimulate the secretion of RANKL by interacting with stromal cells [45], but also sensitized the osteoclast precursor cells to RANKL [46]. Takami et al. reported that high Ca^2+^ could stimulate the secretion of RANKL and induce osteoclastic differentiation in a co-culture of osteoblasts and hematopoietic cells with osteoclastogenic factors free [47]. RANKL produced by myeloma cell itself can directly stimulate osteoclast formation [29, 48]. In agreement with these reports, we found that [Ca^2+^]_o_ up-regulation led to excessive secretion of RANKL in MM cells, which accounted for osteoclastic differentiation in co-cultured systems. Unexpectedly, inhibiting TRPV2 channel activity by SKF96365 abolished high [Ca^2+^]_o_-induced unbalance of the OCL/OBL differentiation and secretion of RANKL. Nevertheless, this result raised the question whether the reduction of osteoclast-related cytokines was due to TRPV2 channel suppression.

Lee et al. expounded that high levels of [Ca^2+^]_o_-induced calcineurin/NFAT signaling activated the secretion of RANKL in osteoblasts [30]. NFATc1 is an osteoclastogenic transcriptional factor and underwent nuclear translocation and auto-amplification with the stimulation of Ca^2+^-calcineurin signaling [49]. The links between TRPV2 and NFAT activity were reported in osteoclastogenesis [50]. Our findings were consistent with previous reports, TRPV2/calcineurin/NFATc3 was significantly increased after treatment with ramp up concentration of [Ca^2+^]_o_ in MM cells, [Ca^2+^]_i_ could induce calcineurin phosphorylation, which in turn led to NFAT dephosphorylation and nuclear translocation [51]. NFAT is known as a key transcriptional factor for RANKL-induced osteoclastogenesis [52, 53]. Unexpectedly, high [Ca^2+^]_o_ could activate calcineurin/NFAT to increase the secretion of RANKL in MM cells, indicating RANKL may be a downstream target of the NFAT. NFATc3 was confirmed to be directly bound to the promoter of RANKL in high [Ca^2+^]_o_. Inhibition of TRPV2 channel in LP-1 cells decreased the affinity of NFATc3 and RANKL. In general, our data revealed that highly efficient stimuli of [Ca^2+^]_o_ could activate calcineurin/NFATc3 pathway through upregulation of TRPV2 in the membrane, subsequently, NFATc3 activation leads to the secretion of RANKL in MM cells via increased NFATc3/RANKL interaction.

SKF96365 has been reported to regulate TRPV2 channel activation-induced calcineurin pathway in brown adipocyte differentiation [54]. SKF96365 dramatically inhibited calcineurin/NFAT pathway and compromised the excessive secretion of RANKL by basal [Ca^2+^]_o_ (0.4 mM) and high [Ca^2+^]_o_ concentration. Furthermore, the inhibitory effect of SKF96365 on osteoclastic differentiation was demonstrated by the reducible expression of TRAP staining and osteoclast marker genes.

## Conclusion

In conclusion, our data showed that TRPV2 overexpression was correlated with poor EFS, OS and bone lesions in MM patients and involved in osteoclastogenesis by activating Ca^2+^-calcineurin-NFATc3 signaling pathway, leading to the excessive secretion of inflammatory cytokines and RANKL, which in turn involved in the progression of osteoclastic differentiation. Here, we uncovered a novel mechanism of MBD, and raised for the first time that SKF96365 could be potential candidate for treatment of MBD.

## Additional files

Additional file 1: **Figure S1.** Schematic model illustrating how TRPV2 regulates calcineurin/NFATc3 signaling pathway and osteoclastic differentiation in high [Ca^2+^]_o_ conditions. **a** The increase of [Ca^2+^]_i_ activates calcineurin/NFAT signalling pathway by TRPV2 channel. Dephosphorylated NFAT is translocated to the nucleus, which leads to increased secretion of RANKL in the bone-marrow microenvironment. RANKL promote osteoclastic differentiation and inhibit osteoblast formation. The inhibition of TRPV2 channel by SKF96365 could reduce secretion of osteoclast-related cytokines and break this vicious cycle in MM. **b** Representative image of TRPV2 expression in NC and MM BM by immunehistochemical staining. (TIF 4337 kb)

Additional file 2: **Figure S2. a**-**j** Point graph depicting the levels of *TRPV2–6* mRNA in MM patients BM plasma cells from the GEO data set (GSE24080). Specimens were divided into groups according to EFS and OS. Microarray analyses showing the *TRPV2–6* expression of MM patients of high/low EFS and OS. **k** The Cancer Cell Line Encyclopedia (CCLE) database showing *TRPV2* expression in cancer cell lines. (TIF 1297 kb)

Additional file 3: **Figure S3. a** Different *TRPV* channels expression levels in 23 MM cell lines from GSE6205. **b**-**f** *TRPV* channels expression levels in NP + MGUS+SMM and MM from GSE5900 and GSE2658. **g** *TP53*, *TRPV2* and *TRPV1* expression levels in LP-1 and U266 from GSE6205. **h** The protein levels of p53 and TRPV2 of MM cells were measured by western blotting. (TIF 1762 kb)

Additional file 4: **Figure S4. a** Double-staining Immunofluorescence detection showing TRPV2 (red) and CD38 (green) in A549 cells. **b** TRAP staining after co-cultures with or without TRPV2 knockdown cells. **c** Cell viability of MM cells treated with high Calcium medium or SKF96365. (TIF 3838 kb)

Additional file 5: **Table1.** Correlations of clinical parameters with serum calcium in 90 MM patients. (DOCX 17 kb)

Additional file 6: **Figure S5. a** The CCK-8 assays of LP-1 incubated with a range of [Ca^2+^]_o_ concentrations. **b** and **c** Western blotting confirming the up-regulation and knockdown of TRPV2 channel in MM cells. **d** Cell supernatants were collected to determine superoxide generation levels. **e** and **f** ELISA showing TNF-α and IL-1β protein expression of LP-1 incubated with a range of [Ca^2+^]_o_ concentrations. **g** The protein levels of calcineurin, nuclear NFATc3 (N-NFATc3) and cytosolic NFATc3 (C-NFATc3) of MM cells were measured by western blotting, Cell fractions of LP-1 over-expressed *TRPV1* were extracted and immunoblotted with antibodies. *P < 0.05; **P < 0.01; ***P < 0.001. (TIF 1893 kb)

## Abbreviations

[Ca^2+^]_i_: Intracellular calcium
[Ca^2+^]_o_: Extracellular calcium
ChIP: Chromatin immunoprecipitation
CTSK: Cathepsin K
EFS: Event-free survival
GAPDH: Glyceraldehyde-3-phosphate dehydrogenase
GEO: Gene Expression Omnibus
IL-1β: Interleukin-1β
ISS: International Staging System
MBD: Myeloma bone disease
MM: Multiple Myeloma
MMP-9: Matrix metalloproteinase-9
MNCs: Multinucleated osteoclasts
NFATc3: Nuclear factor of activated T-cells, cytoplasmic 3
NP: Normal plasma
OS: overall survival
PCR: Polymerase chain reaction
RANKL: Nuclear factor κ B ligand
TNF-α: Tumor necrosis factor–α
TRAP: Tartrate-resistant acid phosphatase
TRPV2: Transient Receptor Potential Vanilloid 2
