# Supplementary figures and images for: TRPV2-induced Ca2+-calcineurin-NFAT signaling regulates differentiation of osteoclast in multiple myeloma
Source: Cell Commun Signal. 2018 Oct 16;16:68. doi: 10.1186/s12964-018-0280-8 (PMC6191893; doi:10.1186/s12964-018-0280-8)

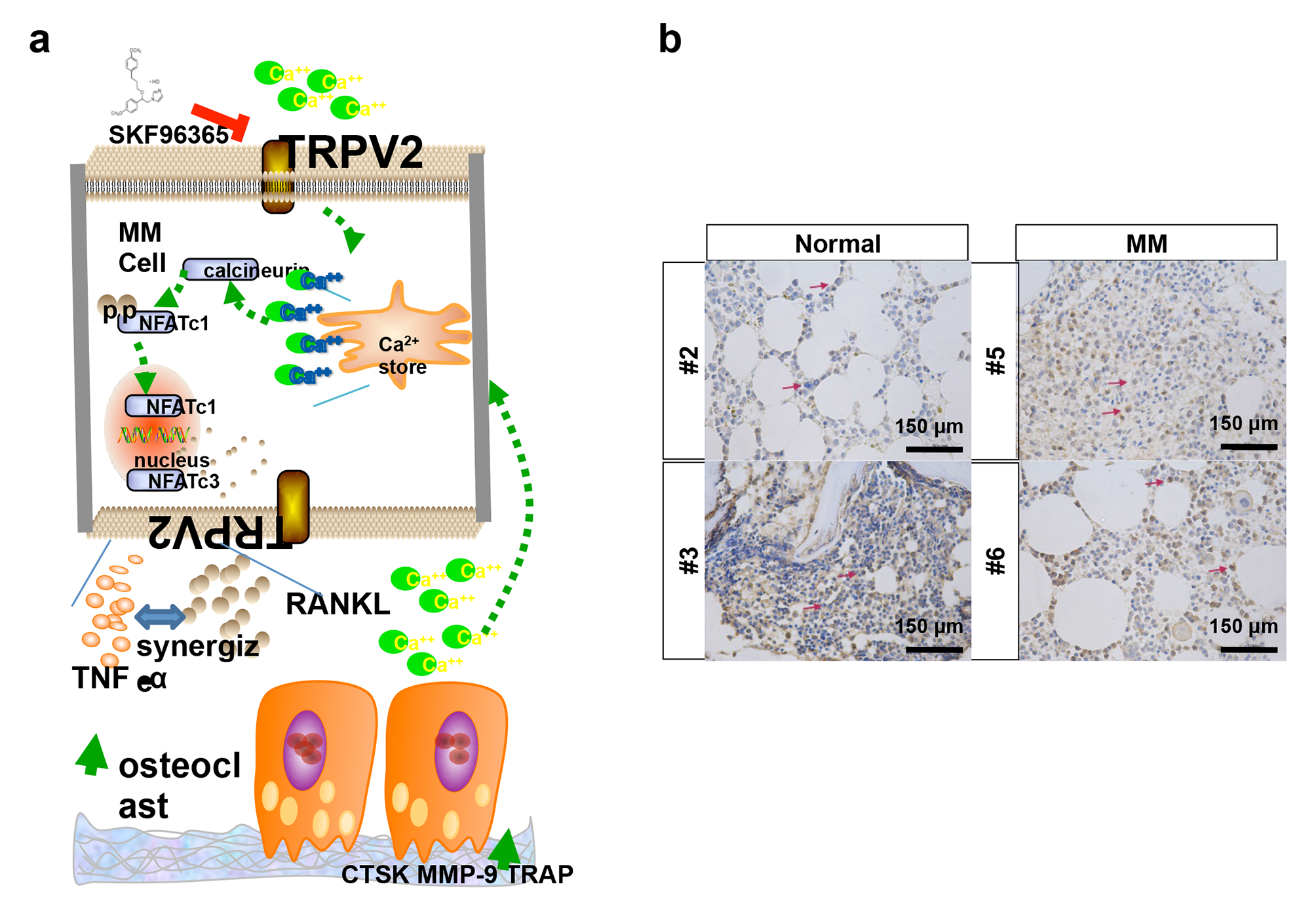

Supplement: Supplementary file 1 — Figure S1. Schematic model illustrating how TRPV2 regulates calcineurin/NFATc3 signaling pathway and osteoclastic differentiation in high [Ca2+]o conditions. a The increase of [Ca2+]i activates calcineurin/NFAT signalling pathway by TRPV2 channel. Dephosphorylated NFAT is translocated to the nucleus, which leads to increased secretion of RANKL in the bone-marrow microenvironment. RANKL promote osteoclastic differentiation and inhibit osteoblast formation. The inhibition of TRPV2 channel by SKF96365 could reduce secretion of osteoclast-related cytokines and break this vicious cycle in MM. b Representative image of TRPV2 expression in NC and MM BM by immunehistochemical staining. (TIF 4337 kb) [file 12964_2018_280_MOESM1_ESM.tif]

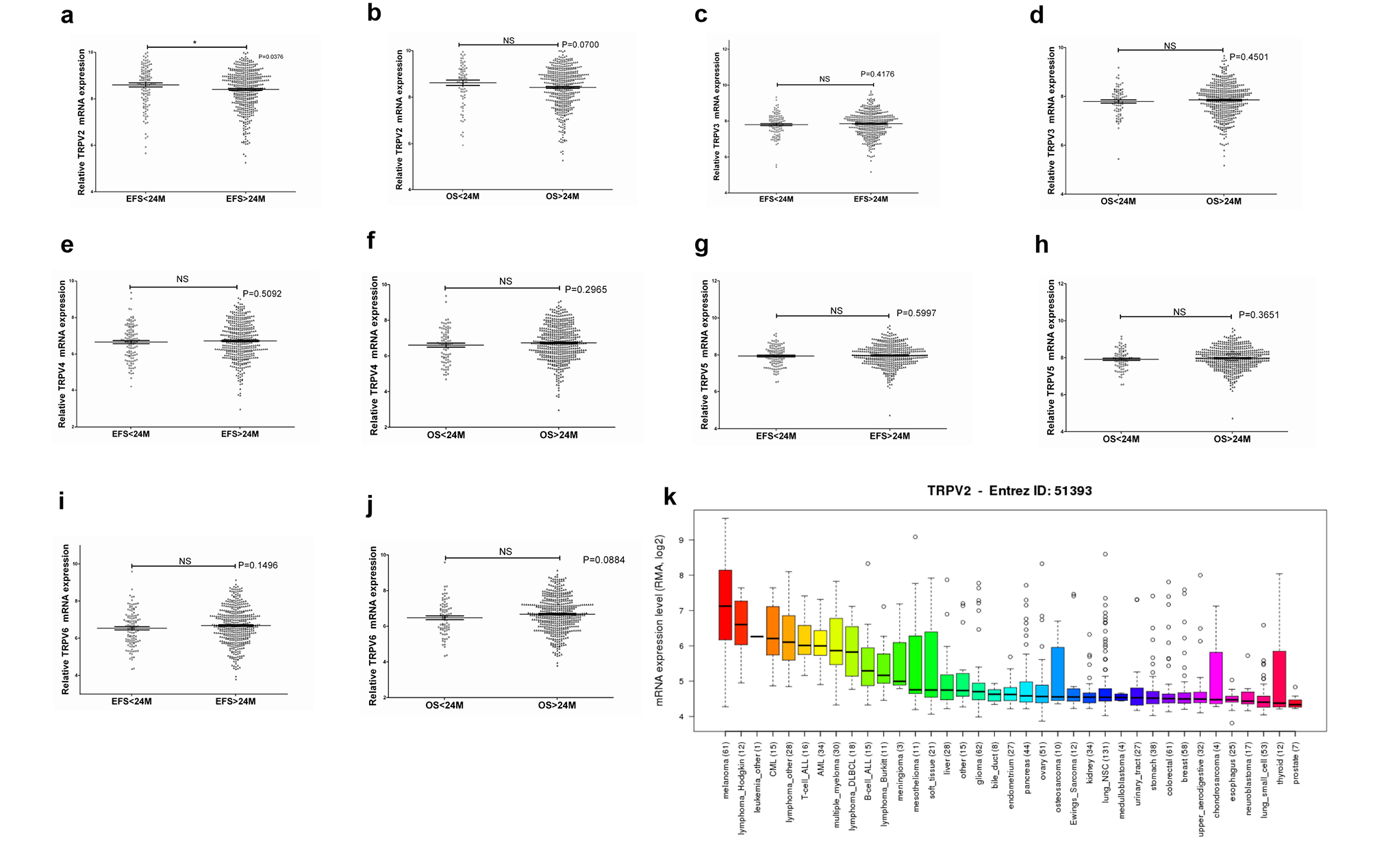

Supplement: Supplementary file 2 — Figure S2. a-j Point graph depicting the levels of TRPV2–6 mRNA in MM patients BM plasma cells from the GEO data set (GSE24080). Specimens were divided into groups according to EFS and OS. Microarray analyses showing the TRPV2–6 expression of MM patients of high/low EFS and OS. k The Cancer Cell Line Encyclopedia (CCLE) database showing TRPV2 expression in cancer cell lines. (TIF 1297 kb) [file 12964_2018_280_MOESM2_ESM.tif]

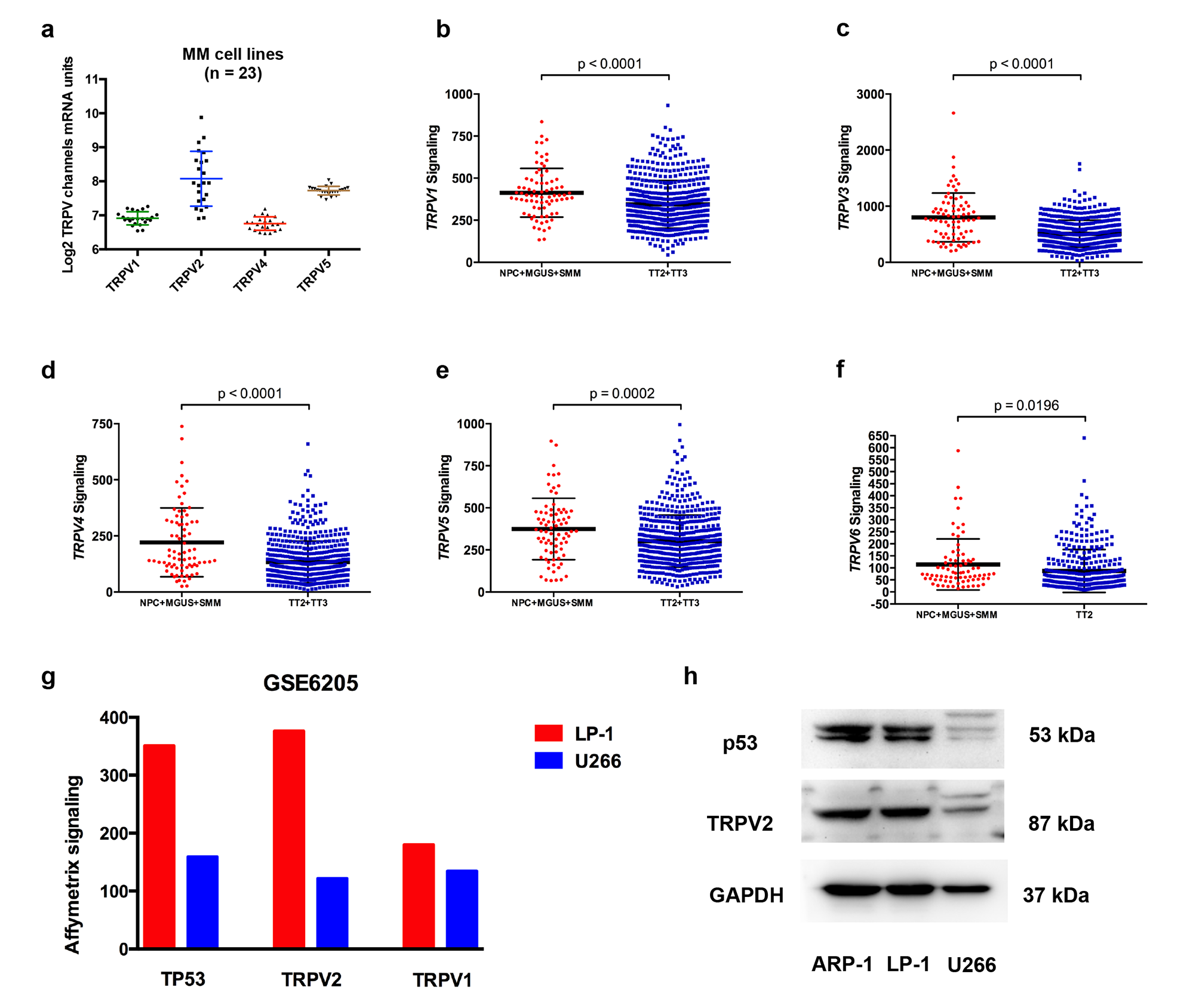

Supplement: Supplementary file 3 — Figure S3. a Different TRPV channels expression levels in 23 MM cell lines from GSE6205. b-f TRPV channels expression levels in NP + MGUS+SMM and MM from GSE5900 and GSE2658. g TP53, TRPV2 and TRPV1 expression levels in LP-1 and U266 from GSE6205. h The protein levels of p53 and TRPV2 of MM cells were measured by western blotting. (TIF 1762 kb) [file 12964_2018_280_MOESM3_ESM.tif]

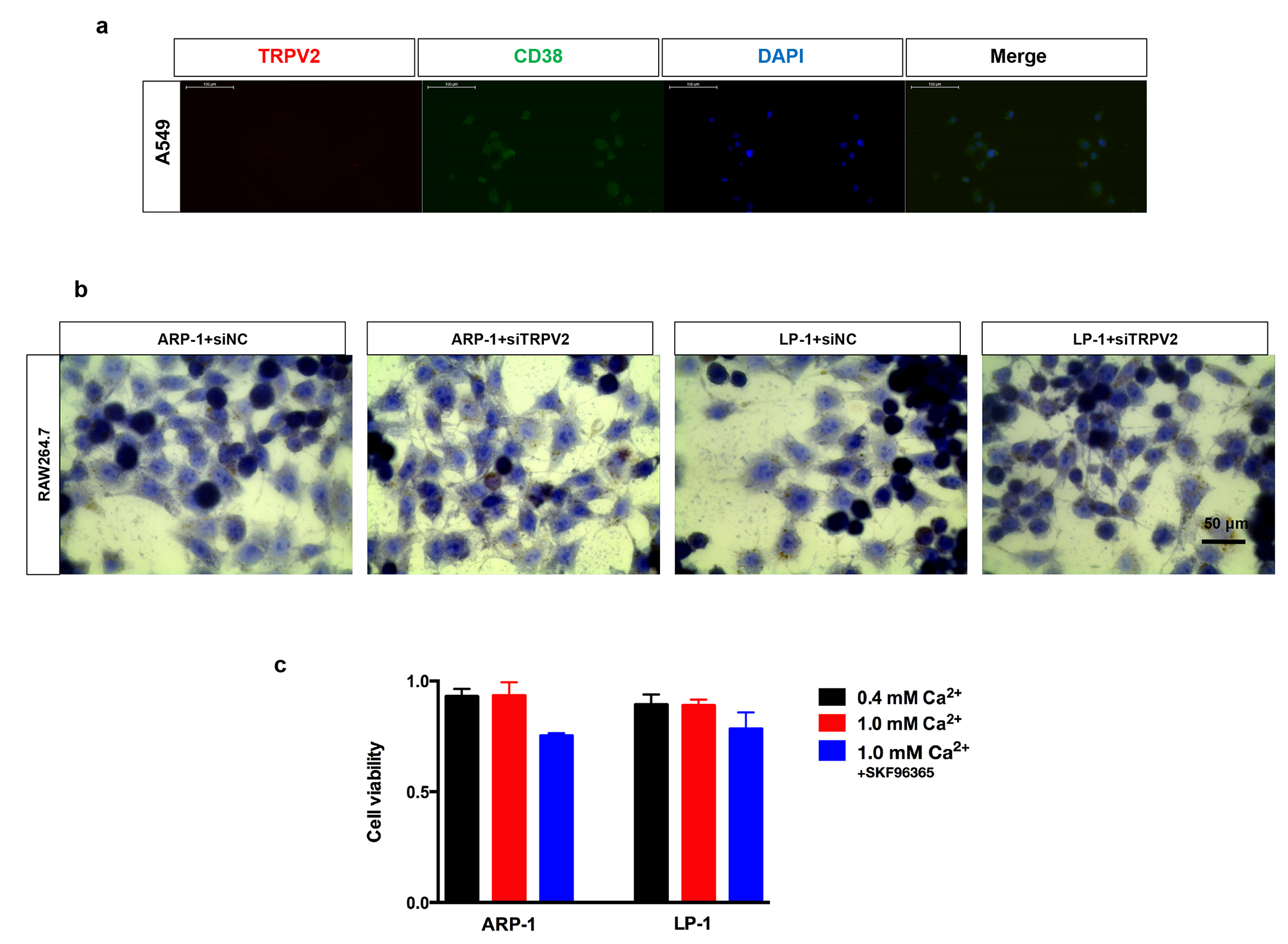

Supplement: Supplementary file 4 — Figure S4. a Double-staining Immunofluorescence detection showing TRPV2 (red) and CD38 (green) in A549 cells. b TRAP staining after co-cultures with or without TRPV2 knockdown cells. c Cell viability of MM cells treated with high Calcium medium or SKF96365. (TIF 3838 kb) [file 12964_2018_280_MOESM4_ESM.tif]

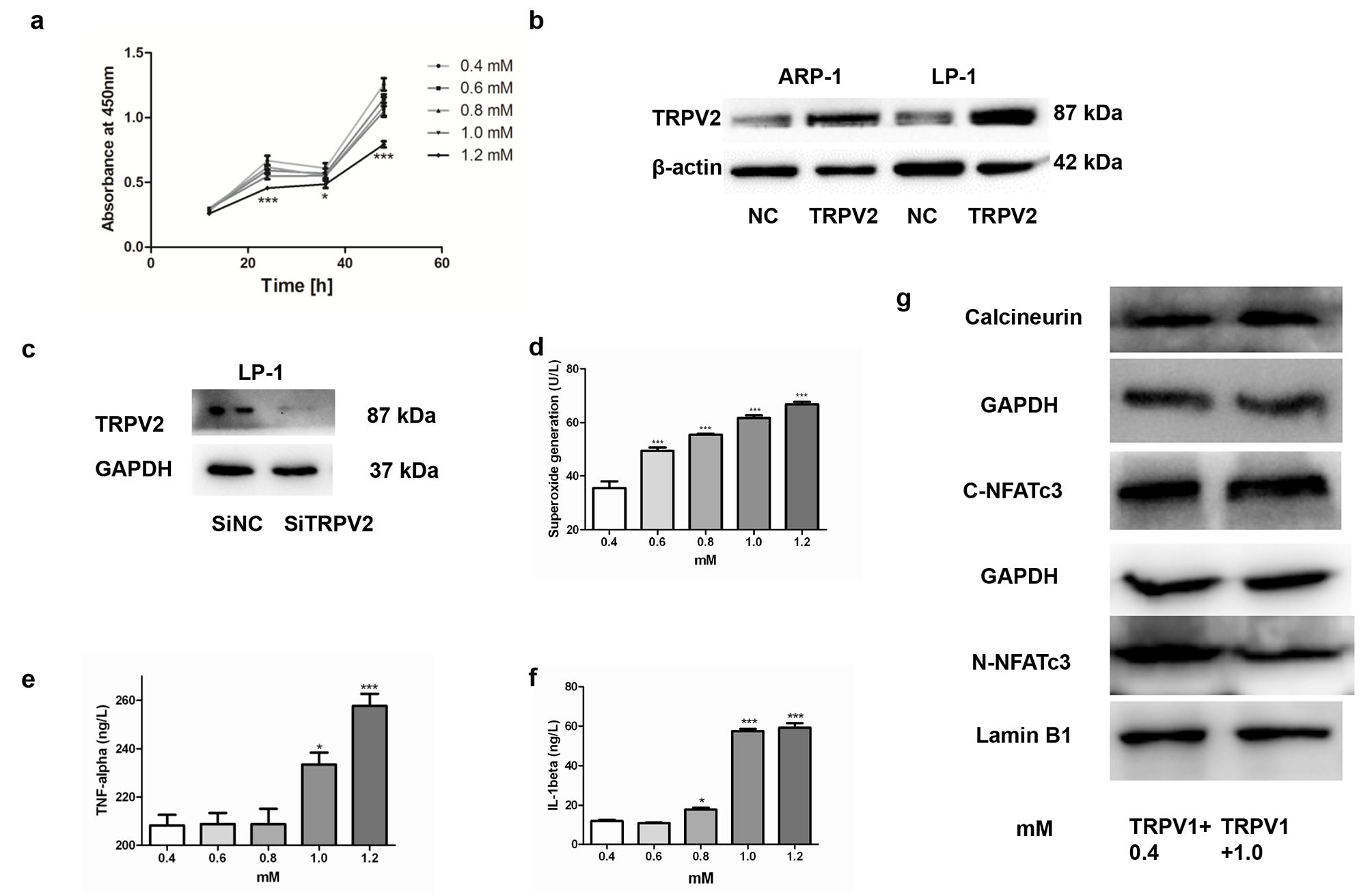

Supplement: Supplementary file 6 — Figure S5. a The CCK-8 assays of LP-1 incubated with a range of [Ca2+]o concentrations. b and c Western blotting confirming the up-regulation and knockdown of TRPV2 channel in MM cells. d Cell supernatants were collected to determine superoxide generation levels. e and f ELISA showing TNF-α and IL-1β protein expression of LP-1 incubated with a range of [Ca2+]o concentrations. g The protein levels of calcineurin, nuclear NFATc3 (N-NFATc3) and cytosolic NFATc3 (C-NFATc3) of MM cells were measured by western blotting, Cell fractions of LP-1 over-expressed TRPV1 were extracted and immunoblotted with antibodies. *P < 0.05; **P < 0.01; ***P < 0.001. (TIF 1893 kb) [file 12964_2018_280_MOESM6_ESM.tif]
