# Supplementary material for: TRPV2-induced Ca2+-calcineurin-NFAT signaling regulates differentiation of osteoclast in multiple myeloma
Source: Cell Commun Signal. 2018 Oct 16;16:68. doi: 10.1186/s12964-018-0280-8 (PMC6191893; doi:10.1186/s12964-018-0280-8)
Supplement: Supplementary file 5 — Table1. Correlations of clinical parameters with serum calcium in 90 MM patients. (DOCX 17 kb) [file 12964_2018_280_MOESM5_ESM.docx]

**Additional file 5: Table S1 Correlations of clinical parameters with serum calcium in 90 MM patients**

| **Clinical index** | | | ***p* value** |
| --- | --- | --- | --- |
| gender | Male (41) | Female (49) | 0.0544 |
| Age (years) | <60 (36) | ≥60 (54) | 0.7179 |
| DS stages | I & II (36) | III (54) | 0.0188 |
| ISS stages | I & II (47) | III (43) | 0.0001 |
| Bone lesions None (41) ≥1 (49) 0.0001  Laboratory examination   \| sCr (μmol/L) \| <176.8 (68) \| ≥176.8 (22) \| 0.0068 \| \| --- \| --- \| --- \| --- \| \| CRP (mg/L) \| <10 (63) \| ≥10 (27) \| 0.5683 \| \| ESR (mm/H) \| <100 (49) \| ≥100 (41) \| 0.0577 \| \| ALB (g/L) \| <35 (46) \| ≥35 (44) \| 0.0025 \| \| LDH (U/L) \| <270 (83) \| ≥270 (7) \| 0.3969 \| | | | |

*p* < 0.05 was considered to reflect statistical significance. Abbrevations: sCr, Serum creatinine; CRP, C-reactive protein; ESR, Erythrocyte sedimentation; ALB, Serum Albumin; LDH, Lactate Dehydrogenase
